# Evaluating the Role of Content in Subjective Video Quality Assessment

**DOI:** 10.1155/2014/625219

**Published:** 2014-01-09

**Authors:** Milan Mirkovic, Petar Vrgovic, Dubravko Culibrk, Darko Stefanovic, Andras Anderla

**Affiliations:** University of Novi Sad, Faculty of Technical Sciences, Trg Dositeja Obradovica 6, 21000 Novi Sad, Serbia

## Abstract

Video quality as perceived by human observers is the ground truth when Video Quality Assessment (VQA) is in question. It is dependent on many variables, one of them being the content of the video that is being evaluated. Despite the evidence that content has an impact on the quality score the sequence receives from human evaluators, currently available VQA databases mostly comprise of sequences which fail to take this into account. In this paper, we aim to identify and analyze differences between human cognitive, affective, and conative responses to a set of videos commonly used for VQA and a set of videos specifically chosen to include video content which might affect the judgment of evaluators when perceived video quality is in question. Our findings indicate that considerable differences exist between the two sets on selected factors, which leads us to conclude that videos starring a different type of content than the currently employed ones might be more appropriate for VQA.

## 1. Introduction

Recently, there has been a tremendous increase in usage of mobile devices to access the Internet and its services. Fierce competition on the end-user electronics market has caused smartphones to become accessible to everyone, and the telecommunications made broadband Internet access cheap and ubiquitous. As a consequence, vast majority of the population in developed countries now owns a cell phone [1], while one quarter of the smartphone owners also possess a tablet [2]. These devices are becoming more potent and versatile by the day, as technological advances make enhanced integrated components (wireless and GPS modules, cameras, different sensors, etc.) affordable and more powerful at the same time. The result of this evolution is not only a change in people's habits when day-to-day tasks are in question, but a shift in the way some traditional services are perceived (such as TV, mail, or telephony). This shift is especially noticeable when video content is concerned, as more and more of it gets consumed “on the move” [3]. In fact, some estimates have it that 86% of the global consumer traffic by 2016 will be generated by streaming video content [4]. Such a high percentage inevitably raises the issue of quality of the delivered content, as perceived by the end-users.

In essence, there are two ways to express the quality of a given video content: the first one is to leverage objective measurements that are inherent to a given sequence in order to derive “objective quality” of the video, while the other is to rely on subjective assessment scores obtained from human evaluators. Ideally, one should be able to use the former to predict the latter. Video Quality Assessment (VQA) algorithms attempt to do this automatically (to assess perceptual degradations introduced by signal processing and transmission operations performed on video sequences and calculate the probable score that should be assigned by human observers), but their performance sometimes leaves much to be desired, and there is considerable room for improvement [5]. In fact, although the objective measurements that algorithms rely on have high reliability and internal validity, it is sometimes questioned if those measurements have useful implications in day-to-day use.

The ground truth data that is used to measure the performance of quality assessment algorithms—which in the domain of VQA takes the form of degraded sequences and Mean Opinion Scores (MOS)—is gathered mostly in laboratory tests on human observers (i.e., naive evaluators are asked to grade various aspects of the presented stimuli). However, there is an array of cognitive biases specific to a human mind, such as contrast error or halo effect [6] which influences the evaluating processes in a way that can jeopardize the planned research designs. These cognitive biases are often content specific, meaning that the content of the presented external stimuli influences the evaluators' judgment when assessing that or another related external stimulus properties [7]. Furthermore, certain video contents can induce high level of emotional activation [8] and that integral emotion (that is content induced) can additionally influence cognition bias, which is dependent on the type of induced emotion [9]. If visual perception process is observed relative to the observer and content, then it makes sense to take an approach that will observe visual perception processes as influenced not only by objectively measurable properties of a stimuli, but also by subjective elements induced by the stimuli within the observer. This user-centered and content-influenced approach in evaluating visual stimuli properties is expected to yield results that can be used when designing future multimedia solutions, as some of the most adaptive delivery mechanisms for streaming multimedia content do not explicitly consider user-perceived quality when making adaptation decisions, in spite of a proven fact that users' nature of perception of adapting multimedia is dynamic [10]. Although some efforts exist when it comes to understanding user experience and leveraging it to remodel the video quality evaluation processes [11], as well as when mobile video delivery is concerned [12], little is said about the effects of presented content on the subjective evaluation [13].

The research presented in this paper aims to define the mental response properties of video stimuli that are pertinent to its content and to propose variables that ought to be taken into account when dealing with the subjective video quality assessments. In order to do that, a standard video quality database commonly used for VQA has been compared to a custom-constructed one based on various content-specific properties that have been shown to influence subjective evaluation such as: user's familiarity with the content and the expectancy of the content (cognitive component), induced emotions (affective component), and user's intent to watch the video again or to recommend it to a friend (conative component). The results indicate a significant difference between the two databases on the observed components and identify the properties of videos that have the highest discriminative power when it comes to subjective video quality perception. In addition, a relationship between video content and the perceived video quality was identified.

The rest of the paper is organized as follows.  Section 2 provides an overview of the relevant published work and current state in the domain.  Section 3 describes the methodology employed and proposed approach in detail.  Section 4 presents experimentally obtained results and provides a discussion.  Section 5 contains conclusions and an outline of possible future research directions.

## 2. Related Work

### 2.1. Sequences Commonly Used in Video Quality Assessment Research

A recent survey by Winkler provides a fairly exhaustive list of publicly available video quality databases [14], which consist of video sequences usually used within research community for video quality assessment tasks. The advantage of using these databases is that the researcher gets a set of video sequences (clips) that have been annotated with subjective quality ratings (derived either using one of the standardized methods proposed by ITU-T [15] or by applying a novel method) that are sometimes tedious and/or time-consuming to obtain—as they require a number of human assessors to watch and grade respective sequences. A potential disadvantage is that the content of the sequences in those databases is usually not selected in a manner that takes into account the “human” components, motivation, or emotions induced that might affect the grading outcome; that is, it seems to be selected mostly on the basis of covering different scenarios that are known to bear weight on impairments induced due to the transport stream or coding/decoding process (e.g., complex or detailed backgrounds, high motion or camera movement, etc.). For example, EPFL/PoliMI Video Quality Assessment Database [16], VQEG HDTV Database [17], Poly@NYU Packet Loss (PL) Database [18], and LIVE Video Quality Database [19, 20] all offer compressed videos corrupted (impaired) by simulated transmission over error-prone networks, MMSP 3D Video Quality Assessment Database [21]—besides being the first public-domain database on 3D video quality—focuses on effects of different camera distances, while Poly@NYU Video Quality Databases [18] contains videos with different frame-rates and quantization parameters. The sequences in these databases exhibit different artifacts (such as ringing, blurring and blocking, just to name a few) that have been shown to affect human perception of video quality and they have even been characterized and quantified using different objective parameters such as Spatial Information (indicator of edge energy), Colorfulness (perceptual indicator of the variety and intensity of colors in the image), and Motion Vectors (an indicator of motion energy for video) [14]. However, the level of perceived degradation seems to be a compound effect of different factors pertinent both to the introduced impairments (which can be objectively measured), properties of Human Visual System (HVS), and possibly emotional state of the assessor, sometimes influenced by the presented content.

### 2.2. The Relationship between Content and Video Quality Perception

Even though the majority of video quality models and objective metrics rely heavily on the low level sensory processing of HVS, research has shown that low level models are not sufficient in characterizing the video quality judgments. For example, a study by McCarthy et al. [22] has shown that assessors are willing to accept objectively poor temporal video quality (e.g., low frame-rate) if they are thoroughly interested in the content of the sequence. In the experiment, participants who were soccer (football) fans, rated low frame-rate (6 frames per second) video as acceptable 80% of the time, even though the motion was not fluid. A similar effect to low frame rate was experienced by assessors in another study [23], where the content of videos presented to them was “frozen” occasionally due to simulated network package loss. Participants in this experiment (who observed videos on mobile devices) were not so keen to accept this impairment and isolated it as one of the top reasons for giving low scores to videos where it was prominent. This study, however, used LIVE Video Quality Database videos (which were sometimes described as “dull” by the participants).

When information assimilation is the primary aim of multimedia presentation, content plays an important role in the perception of multimedia video quality, as reported by Gulliver et al. [24]. They discovered that the content of the sequence has a more significant effect on a user's level of information transfer than either the frame rate or display device type. However, when information transfer is left out of the equation, participants in the same study found frame-rate and device type important for perceived video quality, demonstrating that they were able to distinguish between their subjective enjoyment of a video clip, and the level of quality which they perceive the video clip to possess. This implies that there is a relationship between clip contents and user-perceived video quality, but components of the equation leading to a final score need to be evaluated carefully.

Finally, Jumisko et al. [13] show that recognition of the content has an effect on perceived video quality evaluation. They found that video clips which were recognized by the participants were generally given lower ratings compared to unrecognized clips, while interesting contents collected higher ratings compared to ones deemed uninteresting (whether they were recognized or not). It is argued that evaluators with previous knowledge about the genre are more demanding for the acceptability of quality. Not in discordance with similar studies in the field [25], they found that audio component (when available in the experiment) compensated to a fair degree the impairments in the visual part of the video, and at the same time, impairments in speech were found to be very distracting. This is justified by the fact that, pertinent to that particular experimental design, audio contains the relevant information and the visual component only supports it (e.g., music videos and news with the narrating voice in the background).

### 2.3. Factors Influencing Visual Perception

Visual perception may be broadly defined as mental organization and interpretation of sensory information received via individual's sight. Human visual perception, while being relatively objective by its means (the sight) and constant in terms of properties of the object being watched (light, surfaces, and textures), is still often significantly subjective due to various factors residing in the observer's mind. Since perception requires interpretation of any received information and since interpretation is a complex phenomenon that is unique to every individual, it is evident that visual perception is heavily influenced by internal and external subjective factors that are often hard to grasp. Visual perception is, still, not fully understood by contemporary science: there are competing theories that concentrate on different aspects of it and offer explanations that are often in discordance with one other [26]. Furthermore, there is a number of physiological and psychological factors that are to be taken into account when assessing visual perception, spanning from fatigue [27] and substance intoxication [28] to observer's expectations and motivation [29].

Also, some cognitive biases are known to distort our interpretation and assessment of what we see and evaluate [30]: attention bias (focusing only on one part of the information set), choice-supportive bias (taking past choices as templates for the new ones), conservatism (underestimating high values while overestimating low ones), contrast effect (enhancement or reduction of one object's perception as result of comparing with a recent, contrasted object), curse of knowledge (advanced knowledge diminishes ability to perceive something from a common perspective), halo effect (tendency to observe and evaluate latter aspects in the light of formerly observed aspects), negativity bias (more attention is given to unpleasant information than to the pleasant one), recentness bias (the tendency to weigh recent events more than earlier events), and selective perception (where expectations influence perception).

Finally, bottom-up processing is known to be driven by the stimulus presented [31] and some stimuli are intrinsically conspicuous or salient (i.e., outliers) in a given context. Saliency is independent of the nature of the particular task, operates very rapidly and if a stimulus is sufficiently salient, it will pop out of a visual scene. This knowledge has been leveraged to incorporate saliency information into objective image quality metrics [32], as it is reasonable to assume that artifacts appearing in less salient regions are less visible and thus less annoying, than artifacts affecting the region of interest. While this assumption holds when natural saliency is in question (when observers are not given a particular task), there are indications that quality assessment task (i.e., when observers specifically look for impairments) modifies the natural saliency of images, so the masking effect of saliency for distortions in background regions should be taken with some reserve when modeling subjective quality experiments [33].

## 3. Methodology

### 3.1. Defining the Test Set

As related research reveals, assessors are by no means indifferent to the content they evaluate. This has potential implications for real-world applications, since subjective perception of quality of some “neutral” content selected on basis of its suitability for introducing particular impairments to video stream (i.e., standardized VQA databases) might differ from quality perception of content that the assessor is familiar with or interested in and pays more attention to (i.e., content that the assessor regularly consumes, such as TV shows and movies). To test this hypothesis, we created an alternative set of videos for comparison with the commonly used ones, which ought to activate mental responses that are not activated to a greater extent within commonly used VQA databases and should thus reflect more truthfully the perceived quality of real-life video sequences as experienced by the end-users when impaired. We will refer to this set as the proposed set. As familiar video content was shown to sometimes get lower quality rating at the same impairment level than the unrecognized one, we included a number of videos that should be fairly familiar to assessors, instead of focusing solely on content that was supposed to induce different emotions or other mental responses. First, based on the reports of national television service [34] as well as on the results of a survey conducted by a media research group [35], we have identified several categories of TV program that are most often viewed by average citizens of Serbia (as participants were all Serbian citizens). The identified categories were

1. TV series,
2. movies,
3. informative shows,
4. entertainment,
5. news,
6. music shows,
7. sports, and
8. other.

While most of the categories are self-explanatory, the “Informative shows” category comprises content that covers a specific topic or event (e.g., such as shows most often encountered on the “Discovery,” “Travel,” or “Animal Planet” channels). Using television program ratings as a guideline [34], we selected 4 to 6 sequences representative of each category and downloaded them from YouTube (the number varied because some of the categories were more popular than the other with the viewers). Second, we leveraged social networks (Facebook, Twitter, and Google+) as well as YouTube itself to gain insight into what videos (in the public domain) are deemed popular within different communities (global when it comes to YouTube and local when social networks are in question). Videos that were circulating social networks that the authors are a part of, and which were highly popular within the community at the time the experiment was devised (i.e., had a lot of views), were downloaded, assigned to appropriate categories, and included in the test set. All of the videos obtained for the experiment were part of public domain and their usage for scientific purposes was not prohibited. Also, they were downloaded in 480p format (while being already compressed by YouTube using H.264). Finally, we have opted for the LIVE Video Quality Database as a representative source of video sequences commonly used for VQA tasks, since it is widely accepted and cited within video quality assessment scientific community, regularly updated, and devised with automatic VQA algorithms in mind [36]. All 10 video sequences available in the database were used in our test set. To make for a fair comparison, the LIVE Video Quality Database sequences were downloaded from YouTube as well, in the 480p format, rather than using the unimpaired database version. These videos will be referred to as the LIVEVQDB, or “standard” video set. All of the videos used in the experiment were scaled (and cropped when necessary) to a common resolution of 576 × 432 pixels (1 : 3 aspect ratio). The audio component of the sequences was left out (i.e., only visual component remained). All sequences were between 10s and 11s long. Thus, the final test-set of videos comprised 66 sequences in total; 56 of them were classified into one of the aforementioned categories and the additional 10 from the LIVE Video Quality Database were assigned to the “LIVEVQDB” category. The distribution of the final set of videos is provided in Table 1.

### 3.2. Experimental Design and Setup

As the first research goal was to determine if the LIVEVQDB video set is similar in ability to activate mental responses to a set of real-life videos observed by media consumers, an experimental research design with two independent sets was adopted. The two sets were compared on a number of relevant variables that identified level of mental activation. Mental response, in this context, was regarded as any kind of an individual's conscious mental activity that is directly induced by specific visual stimuli presented to that individual. From various classifications of mental activities available, the widest nomenclature was used in this research: the tripartite classification of mental activities (responses) into cognition, affection, and conation. This classification has been in use since the eighteen century and is still widely used nowadays to address various aspects of human mental state and responses [37].

Cognitive mental activities are mostly observed as a “rational,” or “objective” peace of mind. These activities are thought to be responsible for processing information that people get from their sensory systems via attention and memory. In order to measure cognitive response, we propose variables we named “interesting” and “familiar” to measure the potential of a stimulus to attract attention and to assess its novelty or the lack of it. In contrast to the cognitive activities, affective activities (often called emotional reactions) are thought to be very subjective and relative to the person who is receiving certain stimuli. As identified in the western cultures by Ekman et al. [38] and reaffirmed universally by Fridlund et al. [39], there are six basic emotions that people express, independently from their culture and other external factors: anger, disgust, fear, happiness, sadness, and surprise. We have adopted this classification when researching emotional response for the video stimuli, thus obtaining six more variables (one for each of the basic emotions). Conative mental activities are the ones that drive subjects towards certain activities, which means that the conative part of the mind uses cognitive and affective parts to fuel its role. Therefore, it is necessary to observe conative responses in order to fully assess cognitive and affective states. For this component, three variables were designed: “appealing” (denoting the subjective level of attractiveness of a particular video), “watch again” (willingness to watch the video again), and “share video” (willingness to share the video with other people).

A measurement of subjectively perceived video quality was expressed in terms of Mean Opinion Score (MOS), which is calculated as the average score for a video sequence over all assessors. It was measured on a 5-point quality scale, ranging from 1 (bad) to 5 (excellent). Finally, to be able to keep track of content that was recognized by the assessors, a variable “seen before” was introduced. Complete set of variables and scales used to measure them is presented in Table 2.

Experimental setup in general and laboratory setup in particular, closely resembled the one proposed by the International Telecommunication Union (ITU) in their recommendations for VQA tasks [40]. Sequences were presented to assessors on a 20′′ monitor (SAMSUNG S20B300B), which was operated at its native resolution of 1600 × 900 pixels. A custom in-house software was developed that enables playback of the sequences against the neutral gray background and voting on each video (i.e., filling in a questionnaire). For each assessor, video sequence playback order was randomized to avoid any ordering effects. After presenting the assessor with the sequence, the software displays a questionnaire, where the assessor is asked to rate selected features on either a 1–7 scale (1 being the lowest/worst and 7 being the highest/best score, clearly labeled in assessor's language besides the scale) or by selecting appropriate answer (depending on the question). The only exception to this was the MOS scale that ranged from 1 to 5. Since the questionnaire comprised 13 questions and it was thus impossible to replicate the exact ITU proposed setup for single stimulus type of experiments without conducting multiple runs, we have placed the question regarding perceived video quality on top (i.e., it was the first question), therefore, complying to the standard procedure as much as possible. Questions regarding cognitive, affective, and conative components followed. Upon completing the questionnaire, software played the next video. Assessors were allowed to see sequences only once (i.e., replay was not possible nor were there any additional runs). The test consisted of one session of about 45 minutes, including training. Before the actual test, oral instructions were given to subjects, and a training session was run that consisted of three videos and a questionnaire following each one, where the subjects were free to ask any questions regarding the test procedure (including clarification of questionnaire questions). 20 subjects—8 male and 12 female—participated in the test, their age ranging from 20 to 64. None of them were familiar with video processing nor had previously participated in similar tests. All of the subjects reported normal or corrected vision prior to testing.

## 4. Results

After the experiment, a number of different analyses were performed. All of them were conducted with two considerations in mind: (1) we operated with a sample size that could possibly be considered small in comparison to established norms for behavioral sciences but at the same time is considered more than adequate for VQA tasks and (2) we operated dominantly with ordinal level of measurement (for the dependent variables).

### 4.1. Mental Responses Relative to the Video Sets

The data obtained was observed relative to the two video sets: (1) the proposed one and (2) the LIVEVQDB set. Separate dimensions were compared, followed by a comparison of subsequently computed variables for the three mental response categories.

**Figure 1:**
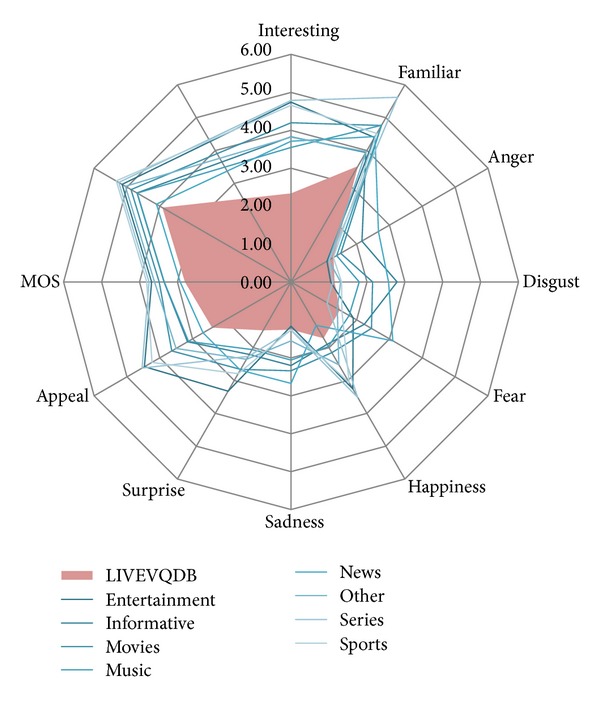
Average scores for different variables (grouped by video categories).

Figure 1 displays average scores over different categories for different variables. LIVEVQDB set is presented in red (filled in area), while videos comprising the proposed set are divided into respective content categories and presented by lines. Just by looking at averages, it seems that videos comprising LIVEVQDB scored significantly lower on almost all measured variables.

To test this hypothesis against the alternative one (that the two observed populations are the same), we ran the Mann-Whitney *U* test, results of which are shown in Tables 3–5. Indeed, the test demonstrated that these sets have very low chance of originating from the same population, suggesting that the proposed set of videos stimulates mental reactions that are significantly more intense than the mental reactions stimulated by the LIVEVQDB set.

As shown in Table 3, the proposed video set achieved higher ranks in both of the cognitive variables observed (“interesting” and “familiar”). A reason that videos comprising this set were found to be more interesting probably lies in their rich and versatile content, which was why they were chosen as good candidates to arouse the attention of assessors in the first place. They were also marked as more familiar to observers than the ones from the LIVEVQDB set, most likely because none of the subjects have participated in VQA experiments before and thus had low odds of encountering videos from LIVEVQDB set before.

The proposed set also achieved higher ranks in all of the six basic emotions observed, as shown in Table 4. This finding is probably a result of original media producer's intent of activating certain affective states, depending on the nature of a video. Conversely, the LIVEVQDB set might have been produced with intention to primarily capture different content that is known to bear weight in terms of introduced impairments when coding/decoding and video transmission is in focus, thus neglecting possible emotional response from the assessors.

The proposed video set was also found to activate conative dimensions of subjects' mental activity to a greater extent than the LIVEVQDB set did. These dimensions are especially important, because they cast a new light on both the cognitive and the affective dimensions. If conative part of one's mind is in idle state, the chances are that the cognitive and affective activation did not happen, even if the subject states the opposite. When somebody is presented with interesting content, he or she is likely to do something after seeing it—either watch it again or share it with relevant others.

The mean ranks difference can be observed in Table 5, but it should be noted that one part of the videos from the proposed set was relatively unpleasant to watch, activating emotions like sadness, fear, anger, or disgust. These dimensions of affective responses were found to be significantly negatively correlated or uncorrelated to the conative dimensions in this research, so the ranks in the conative dimensions would be even higher if we were to select only pleasant and appealing videos, thus showing even greater difference from the standard set.

After individual analysis, the dimensions were summed into the corresponding mental components, forming three variables named cognitive component (comprising 2 dimensions), affective activation (comprising 6 dimensions), and conative activation (comprising 3 dimensions). Again, the Mann-Whitney *U* test has demonstrated significantly higher mental activation induced by the proposed set than the mental activation induced by the LIVEVQDB set (Table 6).

Finally, relationship between the mental activation dimensions and perceived video quality (MOS) was observed. Since all of the dependent variables were of ordinal type, Spearman's Rho measure of correlation was used, following common guidelines of interpretation for behavioral sciences [41, 42]. Moderate correlations, significant at the .01 level (2-tailed), were found between video quality assessment and the following dimensions: interesting (.472), happiness (.415), surprise (.306), appealing (.497), watch again (.393), and share video (.395), while other dimensions mostly correlated with low coefficients.

Also, video quality assessment was correlated with the three calculated mental components, gaining moderate correlation with cognitive component (.412) and conative activation (.496), while low correlation was found with affective activation (.232), again all significant at the .01 level (2-tailed).

Additionally, Spearman's rho correlations were calculated for the three mental components, suggesting a strong correlation between the cognitive and the conative components (.741), while also suggesting a moderate correlation between these two components in relation to the affective component (.351 with cognitive) and (.462 with conative).

### 4.2. Video Quality Assessment

Unknown to assessors, all videos in the full test set were obtained from the same source and with the same quality settings (H.264, 480p) and were hence of roughly the same visual quality. Even though we could not control for the impairments and objective video quality of the source sequences (apart from the LIVEVQDB database), close visual inspection of the obtained material assured us that nonexperts should hardly be able to tell the difference between the quality of sequences presented to them (i.e., any differences they observed would stem from sources not pertinent to visual impairments). To further confirm our assumption, a subset of 20 sequences was played to three independent video quality experts. Among these videos, 10 were randomly selected from the proposed set, while the other 10 were LIVEVQDB sequences (all of them obtained from YouTube to account for any codec effects). Playing order was randomized and the experts were asked to vote only on quality (i.e., they did not take the full experiment) of the videos. Subsequently, unpaired *t*-test was run on MOS obtained and it did not show statistically significant differences between the samples, thus confirming our initial assumptions.

Since a correlation was established between different variables and MOS and LIVEVQDB videos scored constantly lower than the videos from the proposed set, we have tried to further investigate and explain these differences.

On average, videos comprising the proposed set scored a MOS value of 3.47, while LIVEVQDB videos scored 2.80. This is a large difference on a 5-point scale, but it only gets larger when individual components shown to affect perceived quality are considered. For example, when interestingness is observed, best ranked LIVEVQDB video was placed 39th (out of 66 videos in the full set). A comparison between the top 10 ranked “interesting” videos (as deemed by assessors) and the sequences from the LIVEVQDB set reveals a mean difference of 1.34 points (2.80 MOS for the LIVEVQDB versus 4.13 MOS for the proposed set), which was further shown by a *t*-test to be statistically significant (*P* < 0.0001, with 95% confidence interval between 1.04 and 1.64). What is even more interesting is that 7 out of 8 categories (for the proposed video set) found their place on the “top 10 interesting videos.” The only category that did not make it was the “movies” category.

When comparison of top 10 MOS rated videos pertinent to different emotions versus LIVEVQDB videos is concerned, results comply with previously discovered correlations. Thus, when a positive emotion such as happiness was induced by a sequence, it yielded a high quality score (MOS = 3.96). Videos that caused surprise were also ranked as of higher quality (MOS = 3.81), while negative emotions caused video to plummet on the quality scale (sadness (MOS = 2.86), fear (MOS = 3.00), disgust (MOS = 2.86), and anger (MOS = 2.89)). Interestingly, one of the videos from the LIVEVQDB found its place among the top 10 videos that the assessors were angry with.

## 5. Conclusions and Future Research

The most important conclusion we drew from the results obtained in the experiment is that the content of video sequence has a strong impact on activating different cognitive, affective, and conative components within assessors. These components, in turn, have been shown (both by this and previous researches) to play an integral role in VQA tasks, whether assessors are aware of it or not. Thus, we provide a number of recommendations that ought to be taken into account when conducting subjective video quality assessment experiments.

First, video content has to be considered very carefully when trying to measure the perceived video quality, as failing to do so might introduce a bias which will reflect in final MOS obtained. It is thus vital to choose the correct type of content for addressing the research question at hand (at any rate, choose the type of content that is most likely to be encountered by the viewers in real-world conditions). Having this in mind, the “content” variable needs to be monitored whenever possible. Second, since emotions of disgust, fear, anger, and sadness are found to have different impact on conative mental activities than emotions of happiness and surprise, additional attention should be exhibited if conative activities are to be observed as dependent variables (especially when it comes to commercially funded research).

Finally, we have shown that subjective video quality assessment might not be such a reliable measure of video quality if video content is not controlled. This has potential consequences on both the subjective video quality models already devised that do not take content variable into account and automatic approaches (i.e., algorithms) that try to predict MOS based on existing (possibly biased) results.

Since the relationship between video content and perceived video quality was identified, new research directions open up for further investigation. Some of those include identifying and quantifying the strength of this relationship, investigating to what degree can a video be impaired without assessors noticing it (relative to video content), and whether content plays the same role in subjective perception of video quality when sequences are impaired with different levels and types of artifacts. Also, a study at a larger scale might reveal other factors pertinent to different populations (gender-wise, culture-wise, and demographically wise) that impact subjective perception of video quality and should thus be taken into account when VQA tasks are in question.

## Figures and Tables

**Table 1:** Distribution of videos in the test set.

Category name	Number of videos
TV series	4
Movies	4
Informative shows	8
Entertainment	6
News	8
Music shows	6
Sports	5
Other	15
LIVEVQDB	10

**Table 2:** Variables used in the experiment.

Variable name	Scale
MOS	1–5 Likert scale
Seen before	Multiple choice question
Interesting	1–7 Likert scale
Familiar	1–7 Likert scale
Anger	1–7 Likert scale
Fear	1–7 Likert scale
Sadness	1–7 Likert scale
Disgust	1–7 Likert scale
Happiness	1–7 Likert scale
Surprise	1–7 Likert scale
Appealing	1–7 Likert scale
Share	Multiple choice question (recoded to 3 point ordinal scales)
Watch again	Multiple choice question (recoded to 3 point ordinal scales)

**Table 3:** Comparison of cognitive dimensions by the two sets.

	Video set	Mean rank	Sum of ranks	Mann-Whitney *U*	Asymp. Sig. (2-tailed)
Interesting	Proposed	706.91	791737.50	60022.50	.000
	LIVEVQDB	400.61	80122.50		

Familiar	Proposed	684.53	766671.00	85089.00	.000
	LIVEVQDB	525.95	105189.00		

**Table 4:** Comparison of affective dimensions by the two sets.

	Video set	Mean rank	Sum of ranks	Mann-Whitney *U*	Asymp. Sig. (2-tailed)
Anger	Proposed	670.14	750557.00	101203.00	.001
	LIVEVQDB	606.52	121303.00		

Disgust	Proposed	678.64	760071.50	91688.50	.000
	LIVEVQDB	558.94	111788.50		

Fear	Proposed	673.29	754080.50	97679.50	.000
	LIVEVQDB	588.90	117779.50		

Happiness	Proposed	678.66	760100.50	91659.50	.000
	LIVEVQDB	558.80	111759.50		

Sadness	Proposed	677.47	758766.50	92993.50	.000
	LIVEVQDB	565.47	113093.50		

Surprise	Proposed	691.99	775029.00	76731.00	.000
	LIVEVQDB	484.16	96831.00		

**Table 5:** Comparison of conative dimensions by the two sets.

	Video set	Mean rank	Sum of ranks	Mann-Whitney *U*	Asymp. Sig. (2-tailed)
Appealing	Proposed	693.20	776385.50	75374.50	.000
	LIVEVQDB	477.37	95474.50		

Watch again	Proposed	684.10	766196.00	85564.00	.000
	LIVEVQDB	528.32	105664.00		

Share video	Proposed	682.23	764107.00	87653.00	.000
	LIVEVQDB	538.77	107753.00		

**Table 6:** Mental components relative to the two video sets.

	Video set	Mean rank	Sum of ranks	Mann-Whitney *U*	Asymp. Sig. (2-tailed)
Cognitive component	Proposed	703.00	787358.00	64402.00	.000
	LIVEVQDB	422.51	84502.00		

Affective activation	Proposed	701.98	786217.50	65542.50	.000
	LIVEVQDB	428.21	85642.50		

Conative activation	Proposed	695.29	778728.50	73031.50	.000
	LIVEVQDB	465.66	93131.50		
